# Total Body Irradiation for Hematopoietic Stem Cell Transplantation: What Can We Agree on?

**DOI:** 10.3390/curroncol28010089

**Published:** 2021-02-14

**Authors:** Mitchell Sabloff, Steven Tisseverasinghe, Mustafa Ege Babadagli, Rajiv Samant

**Affiliations:** 1 Division of Hematology, Department of Medicine, University of Ottawa, Ottawa, ON K1H 8L6, Canada; msabloff@toh.ca; 2 The Ottawa Hospital Research Institute, Ottawa, ON K1H 8L6, Canada; 3 Division of Radiation Oncology, Gatineau Hospital, Gatineau, QC J8P 7H2, Canada; steven.tisseverasinghe@ssss.gouv.qc.ca; 4 Division of Radiation Oncology, The Ottawa Hospital, Ottawa, ON K1H 8L6, Canada; rsamant@toh.ca

**Keywords:** total body irradiation (TBI), hematopoietic, stem cell transplantation

## Abstract

Total body irradiation (TBI), used as part of the conditioning regimen prior to allogeneic and autologous hematopoietic cell transplantation, is the delivery of a relatively homogeneous dose of radiation to the entire body. TBI has a dual role, being cytotoxic and immunosuppressive. This allows it to eliminate disease and create “space” in the marrow while also impairing the immune system from rejecting the foreign donor cells being transplanted. Advantages that TBI may have over chemotherapy alone are that it may achieve greater tumour cytotoxicity and better tissue penetration than chemotherapy as its delivery is independent of vascular supply and physiologic barriers such as renal and hepatic function. Therefore, the so-called “sanctuary” sites such as the central nervous system (CNS), testes, and orbits or other sites with limited blood supply are not off-limits to radiation. Nevertheless, TBI is hampered by challenging logistics of administration, coordination between hematology and radiation oncology departments, increased rates of acute treatment-related morbidity and mortality along with late toxicity to other tissues. Newer technologies and a better understanding of the biology and physics of TBI has allowed the field to develop novel delivery systems which may help to deliver radiation more safely while maintaining its efficacy. However, continued research and collaboration are needed to determine the best approaches for the use of TBI in the future.

## 1. History

Early in the 20th century, the toxicity from radiation on the human body became evident through accidental exposures [1,2,3]. Subsequently, throughout the 1950s, key figures such as Leon O. Jacobson and Egon Lorenz, while studying the effects of radiation and searching for ways to reverse their detrimental effects, noted in controlled settings with animals that one could salvage the bone marrow with marrow cells from a donor animal after being exposed to radiation [4,5,6,7,8]. This technique was then extended to humans exposed to nuclear accidents [9]. Later, during the 1960s in Canada, Till and McCulloch, while studying the sensitivity of mouse bone marrow cells to radiation, made some of the first observations that “stem cells” could recapitulate the blood system [10].

This knowledge was then developed into the technique of marrow transplantation which could be applied to humans to eliminate marrow disease with radiation, such as acute leukemia, and then salvage the irradiated bone marrow using donor marrow. Many of the techniques and methods that permit the successful engraftment of donor cells, such as extracting “stem cells”, graft versus host disease (GVHD) prophylaxis, human leukocyte antigen (HLA) matching and essential supportive care, were pioneered by Dr. E. Donnall Thomas and colleagues throughout the 1970s—work for which he was award the Nobel Prize in 1990 [11,12,13,14,15,16]. Throughout the decade, they observed that higher doses of radiation would prolong recovery of the bone marrow, which could be salvaged within 2 weeks at a dose of around 1000 roentgens, from opposing ^60^Co sources, with donor marrow and this process did not produce troublesome radiation sickness in patients. They also noted that this dose could produce a remission, although it was not durable. Treating patients with chemotherapy prior to TBI, to reduce the burden of disease, and adding cyclophosphamide to the TBI conditioning, to add both cytotoxic as well as an immunosuppressive effect, resulted in some prolonged remissions [12]. Morbidity and mortality, however, were significant with over half of patients experiencing interstitial pneumonitis, and a third subsequently dying, respectively. With newer chemotherapies in modern times, the number of combinations with TBI has started to flourish in an attempt to maintain the cytotoxic effects of TBI while minimizing associated toxicity [8,17,18,19,20].

## 2. Challenges in TBI Delivery Over the Years

Total body irradiation is based on the principle of delivering a uniform dose of ionizing radiation distributed throughout the body. Ionizing radiation causes cell death through several mechanisms. The most accepted mechanism is the introduction of single and double strand DNA breaks [21]. Radiation dose and cell-killing ability are directly proportional without a maximal effect on cell killing as the dose is increased [22,23].

However, delivering TBI adequately, safely, and efficiently has proven to be both challenging and complex. Even with the modern equipment and tools available today, it requires a sophisticated and coordinated approach, and a highly dedicated team of individuals including radiation oncologists, medical physicists, dosimetrists, nurses, and radiation therapists who are able to communicate and work seamlessly with the transplantation team. Both groups need to understand the underlying intricacies of their respective specialties and it is also far more practical and convenient if the radiation center is physically in the same location as the transplant facility. Furthermore, in contrast to the bulk of radiation oncology which is accustomed to delivering high doses of radiation to a relatively small area in a large number of patients with solid tumors, TBI is focused on delivering a low dose of radiation homogeneously to the entire body in a small number of patients. This clash of philosophies sometimes renders it more difficult to justify the dedicated equipment required for TBI. However, if a coordinated approach is taken and adequate resources are available, it is possible to incorporate TBI into hematopoietic cell transplant programs in a convenient and consistent manner for appropriately selected patients who can benefit from such treatment.

There are several basic approaches to treating patients. The entire body can be treated in one static very large field through multiple junctioned fields and the patient may move through the beam or the beam may sweep across the patient. There are advantages and disadvantages to each approach, but the most important issues are related to patient comfort, reproducibility, and reliability in set-up, consistent and homogeneous dose delivery throughout the body, and the ability to verify the accuracy of the delivered doses.

Factors that affect the final “delivered” dose, as described below, include both patient and machine factors. Patient factors include body size and density, performance status, and ability to maintain a certain position for a period of time. Equipment factors include the size of the treatment room available at a center, treatment planning or dosimetry software, treatment record and verification systems being used, types of machines, delivery techniques, source of radiation, number of fractions, dose rate, and modulation of the delivered dose to certain parts of the body. As radiation treatment technology continues to evolve, advances in new techniques enables fine tuning of the safe delivery of TBI through improved treatment planning approaches, as discussed below [24,25,26,27,28,29].

### 2.1. Patient Factors

A patient’s body habitus and individual density variability pose many challenges to the planning of TBI. The difficulty is to treat a large volume, such as the entire body with its variations in shape, thickness and tissue density, in a uniform manner. Incorporating CT scans into the planning phase has helped to deliver a more uniform dose [30,31]. Even though CT planning for TBI is still not uniformly used, recent studies conclude that dosimetric accuracy and optimization improve with conformal (3-dimensional) planning approaches [32,33] New and improved techniques of imaging the body, in an attempt to ensure a more uniform delivery of the desired dose, continues to be an area of investigation [34,35].

### 2.2. Positioning

Patients need to be positioned in such a way that the beam encompasses the whole body. Often this requires crouching or sitting. Most centers use opposing anterior and posterior fields with the patient standing upright several meters from the source and the beam pointed horizontally. A standing technique is well tolerated and does not disrupt the daily routine use of the machine [24]. Alternatively, patients can be irradiated with lateral fields in a sitting or partly reclining position. This approach is usually better tolerated by patients but presents additional dosimetric challenges that must be considered to improve dose uniformity [36,37].

**Figure 1:**
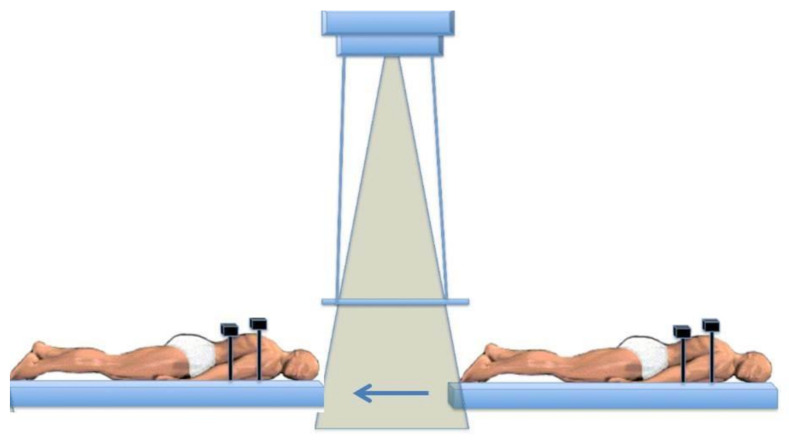
Schematic diagram of a translating-bed technique for total body irradiation (TBI) with the patient being moved through the radiation beam with lung and kidney attenuators in place.

At the Ottawa Hospital Cancer Centre, our patients have been treated on a translating bed that moves under a stationary radiotherapy beam pointing towards the floor [38]. The patient is treated in the supine and then the prone positions under the radiation field (Figure 1). We have generally delivered a dose of 12 Gy (at mid-plane) in 6 fractions (2 Gy/fraction given twice daily) with 10–18 MV photons using a megavoltage linear accelerator at a dose rate of up to 120–140 cGy/min over 3 days. There is always a minimum of 6 hours between fractions. The average time to deliver each fraction is 20 minutes per side (either supine or prone). Then the patient is turned and the rest of the dose is delivered, with an interval of about 20 minutes between the prone and supine fields.

### 2.3. Source of Radiation

Originally, single-fraction radiation with a cobalt machine, originating from research in Canada under the supervision of Dr. Harold Johns, delivered a single treatment field [39]. However, in order to treat the entire body, it was necessary to have the patient positioned sitting or standing sometimes far from the radiation source, and the treatment could last hours because of the low dose rate received by the patient. Patients often developed nausea and vomiting during the actual treatments, which could lead to delays resulting in a prolonged treatment time.

Today, linear accelerators have largely replaced the cobalt machines at most centres [37,40,41]. Linear accelerators simplify and reduce the duration of TBI delivery [42]. Furthermore, interstitial pulmonary syndromes, an early and devastating complication of TBI, appear to have been reduced with the adoption of linear accelerators, although it is uncertain if this is due to the increased flexibility of radiation delivery permitted by linear accelerators compared to the limited options available with the cobalt machines [43]. Newer machines, such as those that use helical tomography, are being studied and deliver radiation throughout the body but can further concentrate the beam on the diseased tissue while greatly sparing the normal sensitive tissues, which may further minimize serious complications from TBI [35].

### 2.4. Fractionations

#### 2.4.1. Single Fraction

Initially, treatment was limited to a single fraction that ranged in dose between 8-10 Gy. This proved to be very convenient for the patient and the most practical method given the equipment available and the techniques initially being used. However, lung toxicity, specifically fatal radiation pneumonitis, was a major concern [42,43,44]. Radiobiology modelling suggests that reduced dose-per-fraction minimizes normal tissue toxicity [45,46]. Although there are limited randomized data to establish the ideal dose and fractionation schedule for use with hematopoietic stem cell transplantation (HSCT), the general principles of radiobiology would suggest reduced toxicity associated with multi-fraction TBI. The general consensus for TBI, as supported by clinical experience, is that there is increased normal tissue-sparing effect using fractionated TBI compared to single fraction treatment for organs including the lungs, liver, eyes, and cartilage [47,48].

#### 2.4.2. Multiple Fractions

The Seattle group were instrumental in identifying that multiple fractions might be safer than a single fraction in TBI [49]. With the introduction of linear accelerators, it became possible to alter many of the variables that are responsible for determining the actual dose delivered to the patient’s tissues. One of these variables was the number of fractions that the total dose could be divided into over a period of time. Fractionation of larger doses was explored and developed with the understanding that the toxicity might be able to be limited while maintaining efficacy [49,50,51,52,53]. From a practical point of view, however, fractionation is more complicated as it requires multiple sessions per day, rendering it more inconvenient.

There has been considerable effort taken to determine the ideal total dose and fractionation regimen to be used. Results have been mixed and conflicting at times, so there continues to be no clear answer. Currently, fractionated TBI doses in the range of 12-15 Gy over 3–4 days are considered reasonable, with 12 Gy in 6 fractions over 3 days, with at least 6 h between treatments, being one of the most common approaches. At our center, as described above, we speculate that we are adding a “mini” fractionation when the patient is flipped from prone to supine during each standard fraction. Therefore, the balance between the duration of radiation, the number of fractions and the amount of time between fractions to allow some tissue recovery continues to be discussed and refined.2.5. Rate of Delivery

Dose rate is a measure of the amount of radiation delivered per unit time. Dose rates are believed to be important and can vary significantly [40]. With the initial cobalt machines, the dose rate was fixed at <5 cGy/min, whereas today with the newer linear accelerators, the dose rate can be adjusted from 10 to >50 cGy/min. Dose rate can influence the biological effect of radiation in terms of improved cell killing and modulating its toxicity on tissues [54]. Although multiple mechanisms may impact the proportion and degree of lung or kidney injury, low dose rates for TBI (<10 cGy/minute) have been favored. Other studies however have suggested that a higher dose rate TBI can be given safely if the total doses are adequately fractionated [52,55,56,57,58]. In Seattle, for instance, it was noted that with lower fractionated doses of TBI, a higher dose rate of 70 cGy/min was required to permit adequate engraftment [59]. Another example might be at our institution where, depending on the machine being used, the dose rate for TBI has consistently been greater than 50 cGy/min, which is much higher than most published series and would be expected to have significant lung toxicity. Yet the rates of clinically significant radiation pneumonitis are below 20% and fatal radiation pneumonitis less than 5% [60]. We presume that this is as a result of fractionation of the total dose and fractionation within each dose during the time the patient is being flipped from prone to supine during the translating-bed technique, as described above, in which the entire body is not radiated at once but rather portions are treated over time. Likely the toxicity is multifactorial and the impact of dose rate alone remains unclear.

### 2.5. Modulation of the Delivered Dose

As mentioned previously, the dose delivered to individual tissues can vary depending on patient factors. The dose can also be modulated along its path in order to treat certain areas more uniformly. Devices such as attenuators and electron scattering spoilers help to modulate the initial dose to in an attempt to minimize toxicity to certain areas such as the lungs and the kidney while also spreading out the beam over the skin, for instance [25,40,61]. Unlike conventional radiation therapy in which skin sparing is often desired, beam spoilers scatter electrons as photons as the TBI beam passes through them, allowing energy to deposit near the surface of the skin in order to capture disease, such as leukemia, that can circulate in the blood volume of the skin [62].

**Figure 2:**
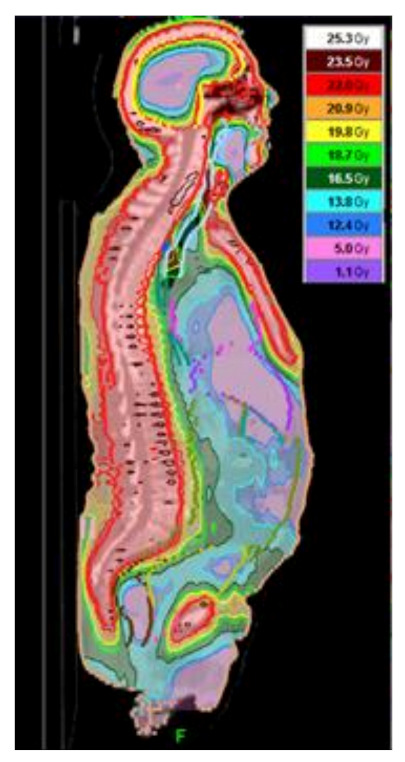
Isodose distribution demonstrating the ability to deliver variable doses of radiation to specific organs with a TMI technique.

Improved imaging techniques, such as intensity-modulated radiation therapy (IMRT), which combines CT imaging with the radiation machine which sends out radiation “wavelets”, allows the software to “sculpt” radiation delivery around certain tissues [63]. This may be an attractive alternative to typical approaches to delivering TBI where the dose delivered is purposely very uniform. Here in Ottawa, we use a similar machine, the TomoTherapy^®^ unit, to deliver total marrow irradiation (TMI) in relapsed myeloma. TMI doses are given twice daily, at a dose of 200–220 cGy per fraction, for up to 5 days (10 fractions). Based on recent dose escalation study, fourteen patients with a median age of 59.5 years received total doses of 14–22 Gy, and at the total dose of 22 Gy to the bone marrow, some of the surrounding tissue only received < 10 Gy (Figure 2) [64]. Adverse events associated with increasing doses of TMI have been primarily in the form of worsening mucositis. Xerostomia was the most common long-term toxicity observed, and it was graded by the LENT-SOMA scale to be ≥ 2 in 63.6%, 38.5% and 25% of the patients at D100, D180 and D365 post-TMI, respectively. There was no difference in length of hospitalization, neutrophil engraftment or incidence of acute toxicity between TMI and the previous autologous transplantation, which was a median of 2.9 years earlier. One patient in the 22 Gy cohort only received 19.8 Gy due to mucositis. Further dose escalation is planned at our centre. Therefore, “targeted” radiation permits the upper boundaries to be raised, potentially enabling better disease control with limited increase in toxicity.

### 2.6. Dose “Delivered”

There are many factors that contribute to the actual dose that is delivered to the patient’s tissues. Unlike the delivery of oral or intravenous chemotherapy, which is straightforward to administer, TBI poses many technical and procedural challenges. After considering the difficulties in measuring the actual doses delivered and considering the very large volumes of tissues treated with TBI, there can still be an inherent 5–10% range of uncertainty or variability in radiotherapy doses being delivered despite all efforts to create a uniform treatment technique. Unfortunately, there has not been a coordinated or concerted effort to standardize TBI. Therefore, as alluded to previously, numerous different approaches are being used today. A recent survey from the European Blood and Marrow Transplant Group demonstrated that there is significant heterogeneity among centers using TBI and this could impact the interpretation of clinical studies [40]. Therefore, it is uncertain whether different TBI techniques will have similar efficacy even when the total doses and fractionation are the same.

## 3. TBI Toxicities

TBI has both short-term and long-term toxicities. Toxicity may be related to various factors including total radiation dose, fractionation, the specific disease being treated, and associated chemotherapy which is combined with radiotherapy, as well as patient-specific factors [65].

In the short term, TBI causes nausea, vomiting, fatigue, mucositis, dysphagia, diarrhea, anorexia, parotitis, xerostomia, erythema, alopecia and myelosuppression.

In the subacute setting, TBI can lead to radiation pneumonitis and sinusoidal obstruction syndrome both of which appear to be dropping in incidence with improvements in TBI delivery, dose fractionation, reduction in lung doses and better supportive care measures [60,66,67,68,69].

Long term toxicity may include cognitive deficits, cataracts, pituitary dysfunction, gonadal failure, hypothyroidism, cardiac dysfunction, xerostomia, osteopenia, dental complications, chronic kidney disease and secondary malignancies. Some of these can be mitigated through shielding or dose adjustment. These toxicities are most concerning in children and young adults, especially with respect to neurologic deficits and future cancer risk. The rates of secondary malignancies due to TBI vary but may increase with higher doses. It is important that patients are closely monitored and counselled on ways to minimize their risk of developing a secondary malignancy, including the role of diet, exercise, alcohol consumption, and abstinence from smoking [70].

## 4. Role of TBI in HSCT: How Does It Compare to Other Regimens?

Although initially it was the only agent used in the conditioning regimen prior to an allogenic hematopoietic cell transplant (alloHCT), today there are many ways of incorporating radiation into the conditioning regimen in order to use it most effectively while minimizing its adverse effects. This can be accomplished by either decreasing its use, decreasing doses, or through techniques to better target the radiation to the appropriate tissue, if possible.

### Methods to Minimize the Dose of TBI

Certain indications for the inclusion of TBI into the conditioning regimen in the past have been challenged over the past decade, either because of new agents that have been able to replace it or studies showing that it no longer provides the advantage that it was once believed to have. Busulfan (BU), as an oral agent, for instance, was combined with cyclophosphamide (Cy) in an attempt to replace TBI, simplifying conditioning in acute myeloid leukemia to a fully chemotherapy-based [71]. This has minimized the indications for TBI in acute myeloid leukemia (AML), avoiding some of its toxicities and hopefully contributing to improved outcomes post-transplant for AML.

Comparisons between BU/Cy and Cy/TBI have been conducted over the years, usually showing superiority for Cy/TBI or equivalence until a recent comparison using BU in its IV form. Busulfan is now almost exclusively administered in intravenous form, and has been demonstrated to be favored or equivalent compared to Cy/TBI when administered in combination with high dose cyclophosphamide in AML, mainly in complete remission [72,73,74]. In acute lymphocytic leukemia (ALL), the necessity of TBI has also been challenged in comparison to a BU IV-based chemotherapy. The MD Anderson group demonstrated a similar outcome to a TBI-based myeloablative (MA) alloHCT for ALL, albeit with different relapse and toxicity profiles [75,76]. However a recent randomized trial in pediatric patients with ALL demonstrated that TBI plus etoposide conditioning resulted in an improved overall survival (OS) and lower relapse risk [77]. Therefore, the conclusion of which regimen is superior continues to be debated and is likely defined by many factors including but not limited to diagnosis, disease status at time of transplantation, other agents in the conditioning regimen and the supportive care received.

Reduced doses of TBI have been explored in the context of a reduced intensity conditioning transplant. Little to no TBI is used to allow engraftment, which may be appropriate in the proper setting due to their potentially lower treatment related mortality [78,79,80,81]. The lower intensity broadens the potential eligible number of patients for an alloHCT, however this must be balanced against their tendency in some cases to result in graft rejection and/or higher relapse rates [82,83,84,85,86].

The Quesenberry group explored a minimal dose of TBI to allow engraftment and found that under appropriate circumstances 1 Gy could allow engraftment of varying degrees. However, the level of chimerism appeared to depend on the intensity of the prior therapy [87]. The Seattle group explored the use of TBI with a dose as low as 2 Gy alongside fludarabine and found that it was sufficient to allow engraftment of donor cells [88]. Diagnosis, disease status, and occurrence of extensive chronic GVHD all influence the outcomes and need to be considered in deciding if this would be an appropriate intensity of conditioning. Others have explored levels between 2 and 8 Gy [81]. This area continues to be explored in order to identify who should and should not receive reduced-intensity conditioning transplantation (RICT) as opposed to a MA alloHCT [89,90]. With novel agents entering the field, the role of transplantation is being re-evaluated in an attempt to further limit patients’ exposure to toxicities associated with such treatments [91].

At the other extreme are groups that are exploring the upper limits of the dose of TBI. Considering poor outcomes associated with higher risk disease, some have advocated for intensifying the conditioning regimen [92,93]. The chemotherapy components have limited room for dose escalation and may already be a large cause of some of the morbidity associated with the transplant [94,95,96,97]. Therefore, TBI may be the most permissible component for consideration of dose escalation. In vitro, there is a direct relationship between the dose of TBI and its cell-killing ability which does not plateau [22,98]. This was attempted over 20 years ago and has been attempted by our centre and others [99,100,101] However, the consistent theme appears to be that relapse is reduced with higher doses, but there is an equal mortality which results in no improvement in overall survival. A recent Center for International Blood and Marrow Transplant Research (CIBMTR) analysis of higher than standard dose TBI compared to the standard TBI dose of 12 Gy also came to the same conclusion [102]. Therefore, trying to capitalize on the beneficial disease control aspects of TBI is worthwhile if the toxicity can be minimized in order to maximize the effectiveness of high-dose TBI in alloHCT recipients.

Beyond straightforward TBI, other strategies have been developed to deliver more targeted radiotherapy. The challenge is to identify the diseased or target tissue. In the case of hematologic diseases, they are usually extensively dispersed throughout the bone marrow spaces and/or the lymphatic system. Studies by the group at City of Hope have explored the technique of total marrow irradiation (TMI), which has been used with or without chemotherapy and has demonstrated promising results [103,104]. This technique can be focused on the marrow environment, where a variety of hematologic diseases reside. More recently they have introduced an alternative imaging technique to better track the location of bone marrow tissue, in order to better target it to further reduce relapse [105]. With this technical advancement in imaging, one can focus radiation on the marrow within the bones, instead of simply focusing the beams on the bones of the skeletal system [105]. Further studies will be needed to understand if narrowing the radiation to this extent will perform as well as traditional TMI.

To further target diseased tissue and spare surrounding normal tissue, radiolabeled monoclonal antibodies have been developed and studied. These antibodies, harbouring a radioisotope, target a specific antigen on the diseased cells. These have been combined with low dose TBI and chemotherapy conditioning to raise the dose locally on the tumour cells. Doses of up to 27, 84 and 24 Gy to the marrow, spleen and liver, respectively, have been utilized [106]. Further studies are ongoing using a radiolabeled antibody for refractory AML [107,108]. Therefore, the options for enhancing or replacing TBI with more targeted radiation therapies are starting to flourish.

## 5. Summary and Future Directions

TBI continues to evolve and has a well-established role as part of the conditioning regimen for hematopoietic stem cell transplantation, though achieving consensus regarding the ideal technique and optimal doses has been elusive. The benefits of TBI have been hampered by its off-target effects, resulting in higher treatment-related mortality. Newer techniques continue to explore methods to minimize these adverse effects in order to realize the disease-controlling potential of TBI. TBI appears to be finding its niche at the extremes of the dose spectrum. At the higher end, it is used to treat disease that has a relatively high risk of relapse. At the lower end, it serves to allow engraftment of donor cells, which permits the immune system to control lower risk disease. Moving forward, further research and collaboration between radiation oncologists and hematologists is required. We need to develop better ways of standardizing techniques in order to be able to reproduce results between centers, and more innovative techniques and technologies that minimize toxicity of TBI are needed in order to realize its maximum benefits, including combining it with targeted drug therapies.
